# Foot posture as a risk factor for lower limb overuse injury: a systematic review and meta-analysis

**DOI:** 10.1186/s13047-014-0055-4

**Published:** 2014-12-19

**Authors:** Bradley S Neal, Ian B Griffiths, Geoffrey J Dowling, George S Murley, Shannon E Munteanu, Melinda M Franettovich Smith, Natalie J Collins, Christian J Barton

**Affiliations:** Pure Sports Medicine, London, UK; Centre for Sports and Exercise Medicine, Queen Mary University of London, London, UK; Department of Podiatry, Faculty of Health Sciences, La Trobe University, Melbourne, Australia; Lower Extremity and Gait studies program, Faculty of Health Sciences, La Trobe University, Melbourne, Australia; School of Physiotherapy, Australian Catholic University, Brisbane, Australia; Department of Mechanical Engineering, Melbourne School of Engineering, The University of Melbourne, Melbourne, Australia; Complete Sports Care, Melbourne, Australia

**Keywords:** Lower extremity, Foot, Pronation, Supination, Prospective studies, Risk factors, Musculoskeletal diseases, Review

## Abstract

**Background:**

Static measures of foot posture are regularly used as part of a clinical examination to determine the need for foot level interventions. This is based on the premise that pronated and supinated foot postures may be risk factors for or associated with lower limb injury. This systematic review and meta-analysis investigates foot posture (measured statically) as a potential risk factor for lower limb overuse injuries.

**Methods:**

A systematic search was performed using Medline, CINAHL, Embase, SportDiscus in April 2014, to identify prospective cohort studies that investigated foot posture and function as a risk factor for lower limb overuse injury. Eligible studies were classified based on the method of foot assessment: (i) static foot posture assessment; and/or (ii) dynamic foot function assessment. This review presents studies evaluating static foot posture. The methodological quality of included studies was evaluated by two independent reviewers, using an adapted version of the Epidemiological Appraisal Instrument (EAI). Where possible, effects were expressed as standardised mean differences (SMD) for continuous scaled data, and risk ratios (RR) for nominal scaled data. Meta-analysis was performed where injuries and outcomes were considered homogenous.

**Results:**

Twenty-one studies were included (total n = 6,228; EAI 0.8 to 1.7 out of 2.0). There was strong evidence that a pronated foot posture was a risk factor for medial tibial stress syndrome (MTSS) development and very limited evidence that a pronated foot posture was a risk factor for patellofemoral pain development, although associated effect sizes were small (0.28 to 0.33). No relationship was identified between a pronated foot posture and any other evaluated pathology (i.e. foot/ankle injury, bone stress reactions and non-specific lower limb overuse injury).

**Conclusion:**

This systematic review identified strong and very limited evidence of small effect that a pronated foot posture is a risk factor for MTSS and patellofemoral pain respectively. Evaluation of static foot posture should be included in a multifactorial assessment for both MTSS and patellofemoral pain, although only as a part of the potential injury risk profile. Whilst the included measures are clinically applicable, further studies are required to determine their relationship with dynamic foot function.

**Electronic supplementary material:**

The online version of this article (doi:10.1186/s13047-014-0055-4) contains supplementary material, which is available to authorized users.

## Background

Identifying lower extremity musculoskeletal injury risk factors is important for sports medicine clinical practice and research, potentially allowing for the development of more effective and efficient prevention and management strategies. Several risk factors have been suggested to increase lower extremity injury risk, including increased body mass index [1], female sex [2] and altered hip mechanics [3].

Foot pronation as a potential lower extremity overuse injury risk factor has received great attention in research and clinical practice. Historically, foot mechanics are considered to contribute to lower extremity malalignment and pathology proximal to the foot via joint coupling with tibial internal rotation [4]. Research has suggested that rearfoot motion (eversion) closely corresponds with tibial motion (internal rotation) [5,6] and is potentially associated with transverse plane rotations at the hip [7]. Based on this model of lower extremity joint coupling, there has long been a theoretical link between foot pronation and lower extremity pathologies including exercise related lower extremity injury, medial tibial stress syndrome (MTSS) and patellofemoral pain [1,8,9]. At the other end of the spectrum increased foot supination has been linked to lower extremity injury via a mechanism of increased limb stiffness and subsequent vertical loading rates [10].

Considering the hypothesised link between foot posture and lower extremity injury, static foot posture is frequently assessed in the clinical setting, with a belief that this may provide indications for biomechanical interventions (e.g. foot orthoses). Commonly employed assessment methods to assess foot posture include, but are not limited to, navicular drop, resting calcaneal eversion, the longitudinal arch angle and the Foot Posture Index (FPI) [11].

Two recent reviews have evaluated the relationship between foot posture and lower extremity injury [11,12]. Tong and Kong [11] concluded that both ‘pronated’ and ‘supinated’ foot types are significantly associated with lower extremity injury, although the strength of this relationship was low, and the authors did not provide a breakdown of individual pathologies or outcome measures. Additionally, this review included studies that were not prospective in nature, which limits the ability to differentiate between cause and effect. Chuter and Janse de Jonge’s [12] narrative review suggested that excessive foot pronation increased the risk of exercise related lower leg pain and MTSS, but not patellofemoral pain. However, this review was not systematic in nature, making conclusions potentially open to bias. Additionally, Chuter and Janse de Jonge [12] focused on dynamic function, and did not include studies related to static foot posture.

To the authors’ knowledge, there has not been a systematic review investigating the relationship between static foot posture or dynamic foot function and lower extremity injury development using only prospectively designed studies. Therefore, the aim of this systematic review was to (i) identify and appraise the current evidence for the prospective link between foot posture and lower limb overuse injury and (ii) provide guidance for future research in this area. This paper, focusing on static foot posture measures, represents the first component of a two-part systematic review on foot function-related risk factors for lower limb overuse injury.

## Methods

The protocol for this systematic review was developed using guidelines provided by the Preferred Reporting of Systematic Reviews and Meta-Analysis (PRISMA) Statement [13] (Additional file 1).

### Search strategy

MEDLINE, CINAHL, Embase, SPORTDiscus and Google Scholar were searched from inception until April 2014. Medical Subject Headings (MeSH) were exploded to encompass relevant subheadings, as well as relevant keywords (Additional file 2). The search strategy limited findings to adult human participants and English language publications. We hand searched reference lists of identified systematic and narrative reviews and contacted field experts (e.g. physiotherapists, podiatrists) regarding known important publications. Additionally, a cited reference search for each included paper was undertaken in Google Scholar.

### Eligibility criteria

A single investigator (GJD) exported all studies identified by the search strategy to Endnote version X5 (Thomson Reuters, Philadelphia). Initial eligibility criteria were: (i) prospective cohort study design; (ii) quantitative measurement of foot posture or function at baseline (static or dynamic); and (iii) prospective collection of specific or non-specific lower limb overuse injury surveillance data over a specified time period. No exclusion was made relative to any given population. Two authors (BSN and IBG) reviewed all abstracts to determine eligibility. Full texts were screened to confirm eligibility, and where there was uncertainty regarding eligibility from the abstract alone. A third reviewer (CJB) was available for any discrepancies.

Studies that fulfilled the initial eligibility criteria were separated into those that investigated static measures of foot posture and those that investigated dynamic measures of foot posture (i.e. measured during walking or running). This review focused on static measures, while dynamic measures are addressed in the accompanying paper [14]. Any study that included both static and dynamic measures of the foot was included, but only data pertaining to static measures was used for this part of the review. Studies that included static foot posture measures that were not quantitative in nature were excluded [15-25]. We defined specific lower limb overuse injuries as those with a single diagnosis and non-specific lower limb overuse injuries as those without a specific diagnosis or where multiple overuse injuries were pooled.

### Quality assessment

The Epidemiological Appraisal Instrument (EAI) [26] was used to evaluate the methodological quality of the included studies. The EAI was designed specifically for cohort studies and consists of 43 items across five domains — (i) reporting, (ii) subject/record selection, (iii) measurement quality, (iv) data analysis and (v) generalisation of results [26]. Individual items were scored as “Yes” (score of 2), “Partial” (score of 1), “No” (score of 0), “Unable to determine” (score of 0) or “Not Applicable” (item excluded). Previous studies have found the EAI to have adequate external validity and good to excellent intra-rater (Kappa coefficient range 52 to 60), and inter-rater (Kappa coefficient = 90% [95% CI; 87-92%]) reliability [26]. The wording of the 43 items was modified slightly for this review to improve clarity and rater interpretation. To maintain validity, no items were removed (Additional file 3).

Two raters (BSN and IBG) who were blind to the author and publication details independently evaluated each study. Discrepancies between the raters were resolved during a consensus meeting. Average scores across the 43 items were calculated, with a maximum possible score of 2.0. Studies were then classified as high quality (≥1.4), moderate quality (1.1 to <1.4), or poor quality (<1.1) [26].

### Data management

Data regarding study characteristics were extracted from each study by two independent investigators (BSN and IBG). This included publication details (year, author, country), participant characteristics (number of participants injured and uninjured, age, sex, eligibility criteria, population [i.e. military]) and study methods (foot posture measurement, examiner details, injury outcome, duration of study, covariates investigated) (Table 1). For continuous scaled foot posture variables means and standard deviations (SD) were extracted for injured and uninjured participants. For nominal scaled variables raw counts of injured and uninjured participants (e.g. injury incidence in categories of foot types) were extracted. Corresponding authors were contacted for additional data if adequate data were not provided in the publication. For studies that described particular foot posture variables but did not publish data, this was recorded as ‘not reported’ (NR) and it was assumed that no significant differences were observed between those who were injured and uninjured.

**Table 1:** **Summary of study characteristics**

	**Population**	**Observation period (activity, duration)**	**Injury outcome**	**Injured group**		**Uninjured group**		**Foot posture measure**
				**N total (n females)**	**Age (mean ± SD)**	**N total (n females)**	**Age (mean ± SD)**	
Bennett *et al*., 2001 [31]	Cross country runners	8 weeks	Medial tibial stress syndrome	15 (13)	15.3 (±1.0)	21 (8)	15.7 (±1.5)	Resting calcaneal position (degrees)
Yates and White, 2004 [32]	Naval recruits	10 weeks basic training	Medial tibial stress syndrome	40 (18)	NR	84 (22)	NR	FPI-8
Burne *et al*., 2004 [33]	Military cadets	12 months	Medial tibial stress syndrome	23 (11)	NR	135 (25)	NR	Resting calcaneal position (degrees)
Willems *et al*., 2006 [34]	Physical education students	12 months	Medial tibial stress syndrome	46 (29)	NR	354 (130)	NR	Resting calcaneal position (degrees)
Reinking, 2006 [35]	Female collegiate athletes	One athletic season	Medial tibial stress syndrome	20 (20)	NR	56 (56)	NR	Navicular drop
Reinking *et al*., 2007 [36]	Collegiate athletes	One athletic season	Medial tibial stress syndrome	60 (31)	NR	28 (13)	NR	Navicular drop
Plisky *et al*., 2007 [37]	High school runners	13 weeks	Medial tibial stress syndrome	16 (11)	NR	88 (29)	NR	Navicular drop
Hubbard *et al*., 2009 [38]	Collegiate athletes	One athletic season	Medial tibial stress syndrome	29 (9)	19 (±0.98)	117 (72)	19.9 (±1.8)	Navicular drop
Bennett *et al*., 2012 [39]	Cross country runners	Cross country season	Medial tibial stress syndrome	26 (13)	NR	NR	33 (15)	Navicular drop
Yagi *et al*., 2013 [40]	High school runners	3 years	Medial tibial stress syndrome	102 (44)	NR	142 (54)	NR	Navicular drop
Hetresoni *et al*., 2006 [41]	Infantry recruits	14 weeks basic training	Patellofemoral pain	61 (NR)	NR	344 (NR)	NR	Resting calcaneal position (degrees)
Thijs *et al*., 2008 [42]	Recreational runners	10 weeks	Patellofemoral pain	17 (16)	39.4 (±10.3)	85 (73)	37.6 (±9.4)	FPI-6
Boling *et al*., 2009 [43]	Naval recruits	1-2.5 years	Patellofemoral pain	40 (16)	NR	1279 (489)	NR	Navicular drop
Beynnon *et al*., 2001 [44]	Collegiate athletes	One college season	Foot/ankle injury	20 (13)	NR	98 (55)	NR	Longitudinal arch angle
Cain *et al*., 2007 [45]	Male Futsal players	One Futsal season	Foot/ankle injury	33 (0)	NR	43 (0)	NR	FPI-6
Winfield *et al*., 1997 [46]	Female marines	10 weeks basic training	Bone stress reaction	12 (12)	NR	89 (89)	NR	Subtalar joint ROM (Goniometry)
Kaufman *et al*., 1999 [47]	Male Navy Seal candidates	2 Years	LL overuse injury	149 (0)	NR	300 (0)	NR	Longitudinal arch angle
Burns *et al*., 2005 [48]	Triathletes	10 weeks	LL overuse injury	37 (NR)	NR	91 (NR)	NR	FPI-8
Rauh *et al*., 2010 [49]	Female marines	13 weeks	LL overuse injury	104 (110)	NR	644 (634)	NR	Longitudinal arch angle
Buist *et al*., 2010 [50]	Novice runners	13 weeks	LL overuse injury	100	NR	476	NR	Navicular drop
Nielsen *et al*., 2014 [51]	Novice runners	12 Months	LL overuse injury	252 (NR)	NR	478 (NR)	NR	FPI-6

LL = lower limb; NR = not reported; FPI = foot posture index.

### Statistical methods

Inter-rater reliability of EAI scores between the two raters was evaluated descriptively using percentage agreement. Differences between scores for “Yes”, “Partial”, “No”, and “Unable to determine” were calculated, with perfect agreement indicated by zero difference. Ratings for the “not applicable” response were excluded from analysis, as no rater interpretation was required.

Extracted means and SD’s for continuous scaled variables were used to calculate standardised mean differences (SMD) with 95% confidence intervals (CI’s). Extracted nominal scaled data was used to caclulate risk ratios (RR) with 95% CI’s. Data for men and women were analysed separately where this information was provided. Data for right feet only were entered when studies provided a breakdown for both feet, to maintain independence of data [27]. All analyses were completed in Review Manager 5.0 (The Cochrane Collaboration, Copenhagen, Denmark). Meta-analysis (data pooling) was performed where homogeneity between studies was deemed to be adequate (i.e. outcome measures were performed and reported in a similar fashion for the same pathology). The level of statistical heterogeneity for pooled data was established using *I*^2^ statistics and associated p values (heterogeneity defined as *I*^2^ > 50%) [28].

Calculated individual or pooled SMDs were categorised as small (≤ 0.59), medium (0.60 to 1.19) or large (≥ 1.20) [29]. A RR > 1.0 indicated that the lower limb overuse injury was more likely to be found in participants with the risk factor present. A small effect was indicated by a RR ≥ 2.0, and a large effect ≥ 4.0 [29]. Effects were considered to be statistically significant if the associated 95% CI for SMD did not contain zero, and the 95% CI for RR did not contain one.

### Evidence-based recommendations

Based on previous work by van Tulder *et al.* [30], levels of evidence were assigned for each foot posture measure evaluated, incorporating statistical outcomes and methodological quality of included studies.

#### Strong evidence

Pooled results derived from three or more studies, including a minimum of two high quality studies that are statistically homogenous; may be associated with a statistically significant or non-significant pooled result.

#### Moderate evidence

Statistically significant pooled results derived from multiple studies that are statistically heterogeneous, including at least one high quality study; or from multiple moderate quality or low quality studies which are statistically homogenous.

#### Limited evidence

Results from one high quality study or multiple moderate or low quality studies that are statistically heterogeneous.

#### Very limited evidence

Results from one moderate quality study or one low quality study.

#### No evidence

Pooled results insignificant and derived from multiple studies regardless of quality that are statistically heterogeneous.

## Results

### Search results

**Figure 1:**
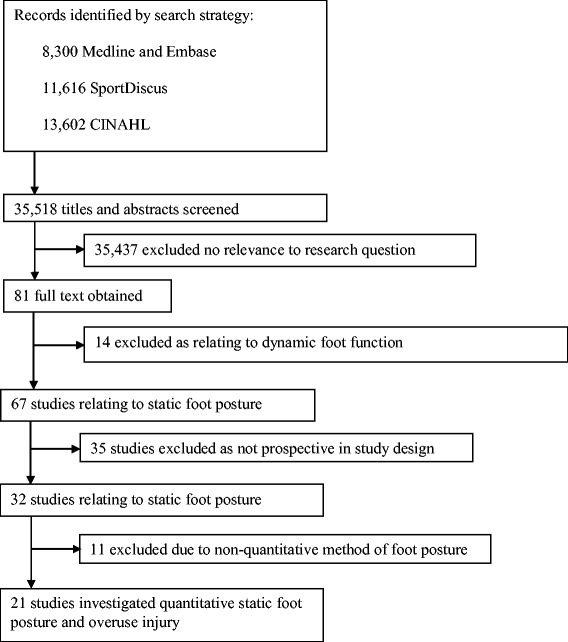
**Search results throughout the review process.**

The electronic database search yielded a total of 33,518 citations across the two parts of this systematic review (static foot posture and dynamic foot function). Following the sequential review of titles, abstracts and full texts, as well as removing studies that were not prospective cohort studies, 32 studies that evaluated static measures of foot posture were identified [15-25,31-51] (Figure 1). Full text versions of these were assessed for eligibility based on static foot posture assessment, and 21 studies met the eligibility criteria [31-51], which were grouped according to injury type.

### Quality assessment of included studies

Based on EAI evaluation, quality scores ranged from 0.8 to 1.7 (out of a possible score of 2.0), with the majority of studies included in this review being of moderate quality (MQ) (n = 13, 62%) [31-34,36,40,42-45,47,49,50] (Additional file 4). Five studies (24%) were classified as high quality (HQ) [35,37,39,48,51], and three studies (14%) as low quality (LQ [38,41,46]). In terms of inter-rater reliability across 35 items included in the quality assessment, 14 items had perfect or near perfect agreement. That is, these items were awarded the same score or there was a maximum of one point difference in scoring. For a further 15 items, the raters had near perfect agreement for >80% of the studies reviewed. Item 35 (‘is prior history of disease and/or symptoms collected and included in the analysis’) displayed the lowest agreement, with perfect or near perfect agreement for only 11 of 21 studies. Percentage agreement across the 35 items ranged from 33% to 100%.

Common themes relating to categories of methodological quality were identified using the EAI [26]. High quality studies scored well for relevant descriptions (e.g. hypothesis, risk factors, participants), statistical parameters and result reporting, as well as adherence to prospective methodology. Poor quality studies generally failed to perform a power calculation with regards to sample size [31-36,38,40,42-50]; demonstrated inadequate or absent reporting of reliability and validity, both for outcome measure [31-34,38,40-42,44-47,49-51] and injury determinant [31-36,38,39,41-46,48-50]; inadequate or absent description of intrinsic and extrinsic variables [39,41,43,46,47]; and inadequate adjustment for these variables [31,32,36,39,41-44,46,47].

### Study characteristics

#### Foot posture variables as risk factors for lower limb overuse injuries

The 21 included studies incorporated a total of 6,228 participants. The participant population varied, with ten studies investigating recreational level runners [34-38,40,42,44,50,51], seven studies investigating military personnel [32,33,41,43,46,47,49], two studies investigating cross-country runners [31,39], and single studies investigating futsal players [45] and triathletes [48]. The types and incidence of lower limb overuse injury were: MTSS 11.5% to 44.1% [31-40,49]; patellofemoral pain 3.0% to 15.7% [41-43]; foot and ankle injury 16.9% to 32.0% [44,45]; bone stress reaction 11.5% [46] and a pooled group of non-specific lower limb overuse injuries 13.9% to 37.5% [47-51].

#### Outcome measure of choice varied

Eight studies investigated navicular drop [35-40,43,50], five studies investigated the foot posture index [32,42,45,48,51], four studies investigated resting calcaneal position [31,33,34,41], three studies investigated the longitudinal arch angle [44,47,49] and one study investigated subtalar joint goniometry [46].

### Medial tibial stress syndrome (MTSS)

**Figure 2:**
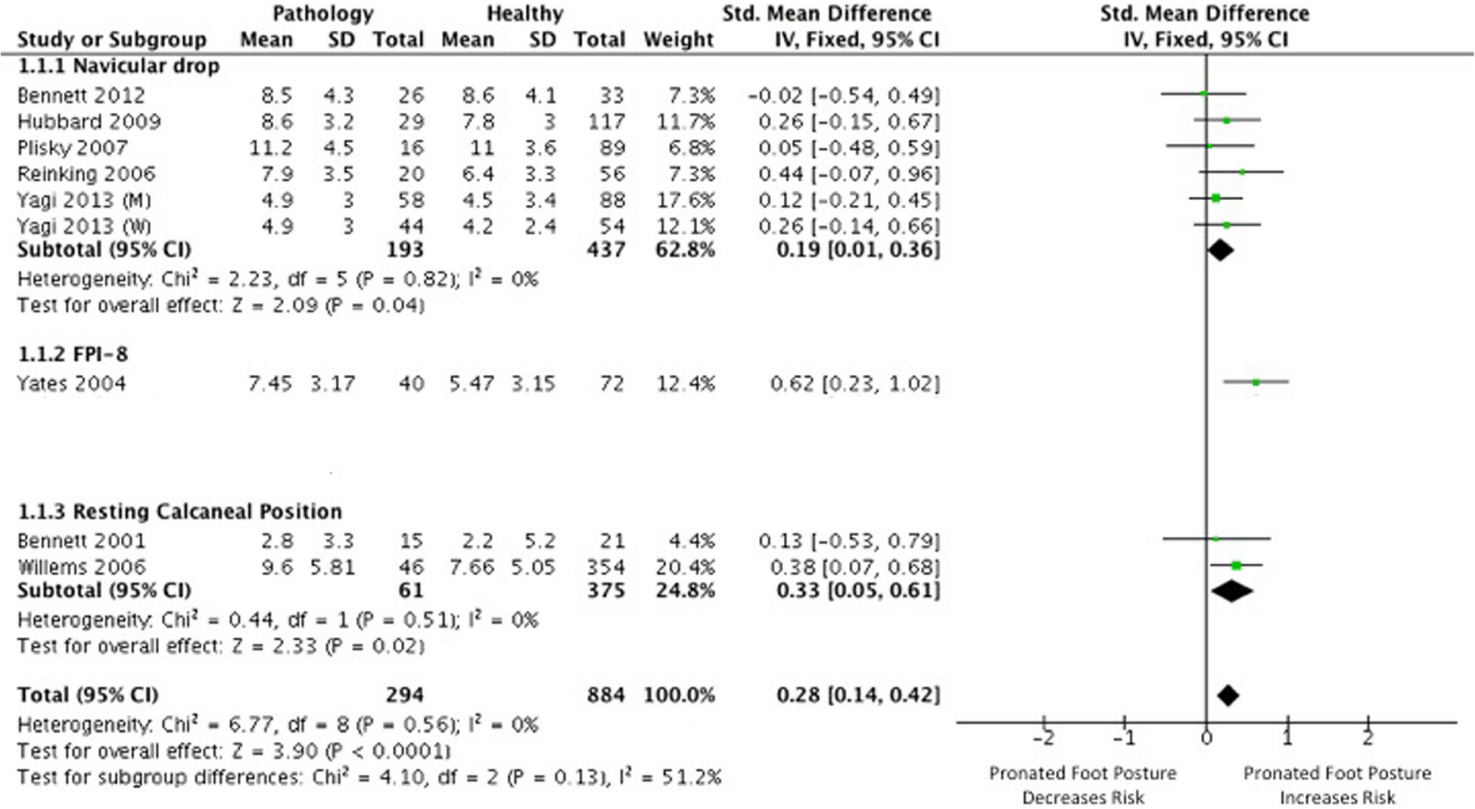
**Forest plot detailing SMD for medial tibial stress syndrome.**

**Figure 3:**
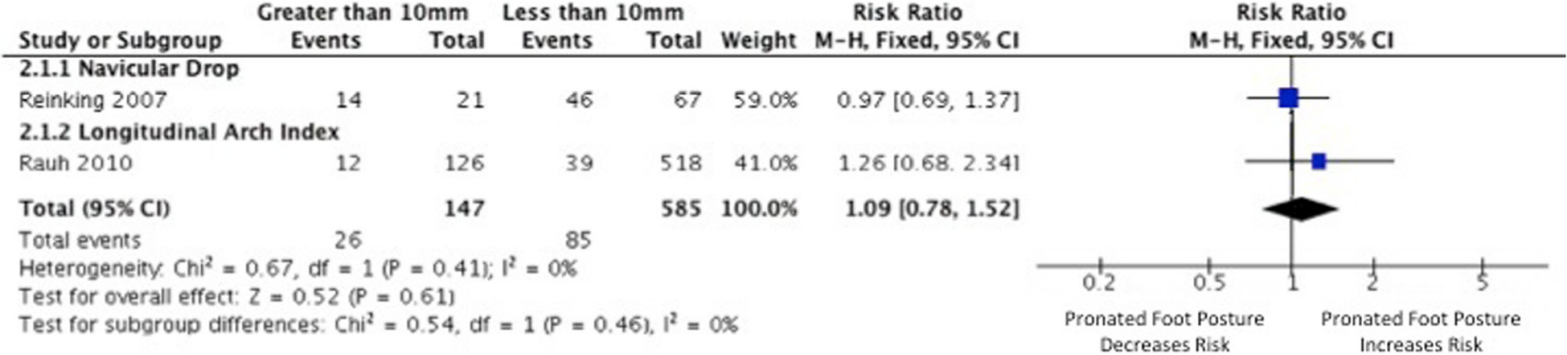
**Forest plot detailing RR for medial tibial stress syndrome.**

Eleven studies [31-40,49] investigated foot posture as a risk factor for the development of MTSS, nine of which provided data suitable for meta-analysis [31,32,34-40,49]. Strong evidence from continuous scaled measures of foot posture (including navicular drop, calcaneal eversion and FPI) indicated that individuals exhibiting a more pronated foot posture were more likely to develop MTSS (3 HQ [35,37,39], 4 MQ [31,34,36,40] and 1 LQ [38]), with a small but significant pooled SMD (I^2^ = 0%, *p* = 0.56, SMD 0.28, 0.14 to 0.42) (Figure 2). When stratifying for foot posture measure, a significant risk association was seen for all three measures, including the FPI (very limited evidence, medium SMD 0.62, 0.23 to 1.02), calcaneal eversion (limited evidence; I^2^ = 0%, *p* = 0.51; small SMD 0.33, 0.05 to 0.61), and navicular drop (Strong evidence; I^2^ = 0%, *p* = 0.82; small SMD 0.19, 0.01 to 0.36). Limited evidence from pooled dichotomous measures (2 MQ [36,49]) indicated no association between foot type (defined by navicular drop magnitude > 10 millimeters) and increased risk of MTSS development (RR 1.09, 0.78 to 1.52) (Figure 3).

### Patellofemoral pain

**Figure 4:**
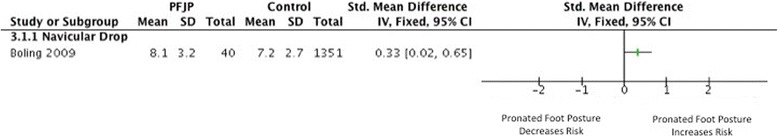
**Forest plot detailing SMD for patellofemoral pain.**

**Figure 5:**
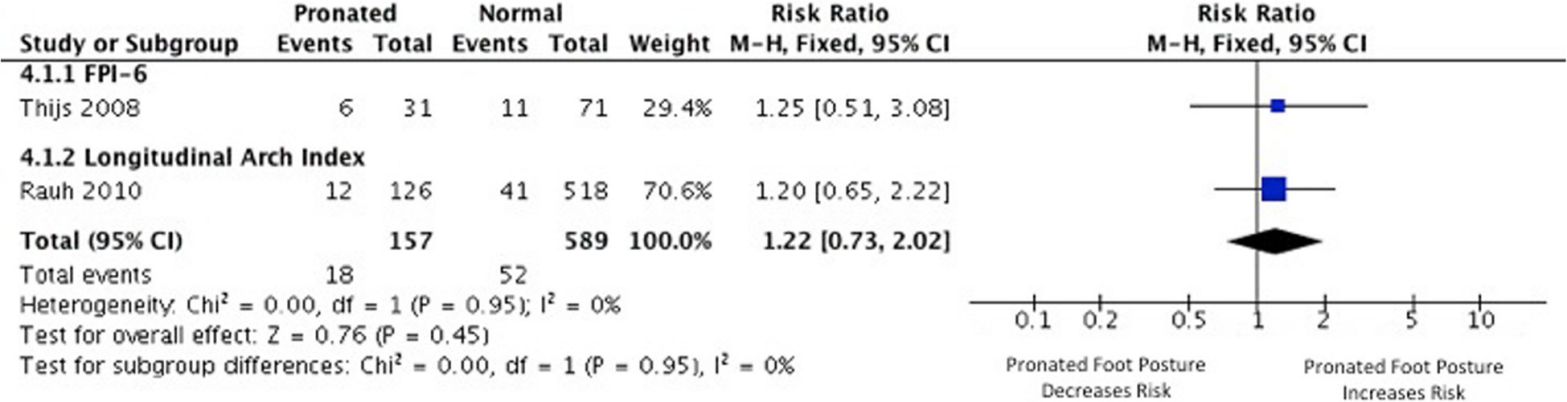
**Forest plot detailing RR for patellofemoral pain.**

Four studies [41-43,49] investigated foot posture as a risk factor for the development of patellofemoral pain, three of which provided data suitable for effect size calculation [42,43,49]. Very limited evidence from continuous measures indicated that individuals exhibiting increased pronated foot posture measured using navicular drop are more likely to develop patellofemoral pain (1 MQ [43]), with a small SMD (0.33, 0.02 to 0.65) (Figure 4). Limited evidence from pooled dichotomous measures (2 MQ [42,49]) indicated no association between a pronated foot posture (defined by FPI and navicular drop) and increased risk of patellofemoral pain development (RR 1.22, 0.73 to 2.02) (Figure 5).

### Foot/ankle injury

**Figure 6:**
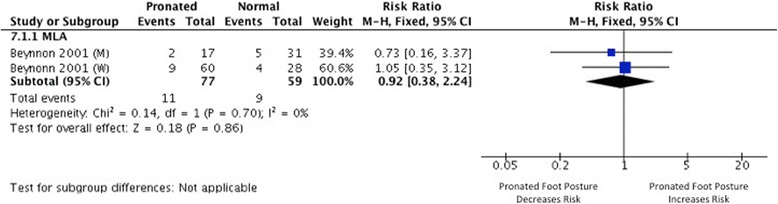
**Forest plot detailing SMD for foot and ankle injury.**

Two studies [44,45] investigated foot posture as a risk factor for the development of foot/ankle injury (defined as any tissue damage, pain and/or physical complaint of the ankle affecting performance or limiting sporting participation [45]). One study provided data suitable for risk ratio calculation [44] (Figure 6). Very limited evidence from dichotomous measures (1 MQ [44]) indicated no association between foot posture (defined by longitudinal arch angle) and increased risk of foot/ankle injury development (RR 0.92, 0.38 to 2.24).

### Bone stress reaction

One study investigated foot posture as a risk factor for the development of bone stress reaction, using subtalar joint goniometry [46]. This measured the gross total range of rearfoot motion from supination to pronation in a non-weight bearing position, with a higher available range indicating increased static pronation. We were unable to calculate effect sizes due to inadequate data reporting.

### Non-specific lower limb overuse injury

**Figure 7:**
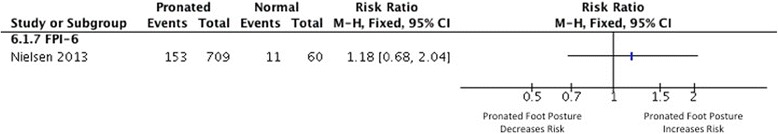
**Forest plot detailing SMD for non-specific lower limb overuse injury.**

Four studies investigated foot posture as a risk factor for non-specific lower limb overuse injury [47,48,50,51], two of which provided data suitable for effect size calculation [48,51]. Limited evidence from continuous measures indicates no association between individuals exhibiting a more pronated foot posture (defined by FPI) and injury development (1 HQ [48], SMD −0.50, −2.28 to 1.28). Limited evidence from dichotomous measures (1 HQ [51]) found no association between a foot posture (defined by FPI) and increased risk of non-specific lower limb overuse injury (RR 1.18, 0.68 to 2.04) (Figure 7).

## Discussion

This is the first systematic review and meta-analysis of prospective research regarding static foot posture and its relationship to lower limb overuse injury development. Findings showed that a pronated foot posture was a risk factor for the development of both MTSS and patellofemoral pain. However, associated effect sizes were small, indicating this relationship is weak and only a part of the multifactorial etiology.

Across the 21 studies included in this review, four different measures of static foot posture were employed, including navicular drop (n = 9), the FPI (n = 5), calcaneal eversion (n = 4) and the longitudinal arch angle (n = 3). Interestingly, the findings of this review, which link foot posture measured using navicular drop and FPI to injury risk (MTSS and patellofemoral pain respectively), are in conflict with reported findings from two studies that were not included within the meta-analysis due to an absence of adequate data [33,41]. Specifically, calcaneal eversion was reported to be unrelated to both MTSS [33] and patellofemoral pain [41] risk. These conflicting findings may be explained by the varying method of foot posture measurement, and the inferior reliability of the calcaneal eversion measurement compared to navicular drop and the FPI [52,53]. They also indicate that calcaneal eversion may be less sensitive in identifying risk of lower limb injury development when compared to the FPI and navicular drop. Further prospective research concurrently collecting multiple foot posture measures is needed to confirm this.

Direct comparison of findings with the review of Tong and Kong [11] is difficult, due to analogous data being unavailable within their results. They concluded that both ‘high arched’ (supinated) and ‘flat foot’ (pronated) types are risk factors for lower extremity injury, but did not provide a breakdown for individual pathologies or outcome measures. Our findings are in agreement with the MTSS systematic review of Newman *et al*. [54], which reported greater navicular drop magnitude to be a risk factor (SMD = 0.26 for continuous scaled data; risk ratio = 1.99 for nominal scaled data). Two additional studies completed by Reinking and colleagues [35,36] are included in our review, which may explain the small variance in our statistical findings (SMD = 0.19) compared to Newman *et al*. (SMD 0.26) [54]. Importantly, these additional findings provide further confirmation of a relationship of small effect between greater navicular drop and risk of MTSS.

Limited and very limited evidence indicated that static foot posture may not be a risk factor for the development of a pooled group of non-specific lower limb overuse injuries or foot and ankle injuries, respectively. In these cases the broad and ambiguous definitions of pathology may have made determining precise relationships with foot posture difficult. Rather than combining all lower limb overuse injuries in analyses, future studies should prioritise evaluation of discrete, well-defined conditions, which will enable more accurate identification of foot posture risk factors for specific injuries.

### Clinical implications

Although a relationship between a pronated foot posture and greater risk of MTSS and patellofemoral pain was identified, the associated pooled SMDs indicate a small effect (0.28 and 0.33, respectively). Therefore, whilst a pronated foot posture may provide an indication of injury risk, other factors should also be considered. Both MTSS and patellofemoral pain are considered to have a multi-factorial etiology [54,55]. It is important that clinicians consider additional established risk factor variables such as altered hip kinematics [3,56], increased body mass index [1] and limited running experience [54] in evaluating possible risk factors.

Another possible reason for the limited relationship between foot posture and injury risk may be the limitation of static measures to predict dynamic function. This has been the subject of much research, with differing conclusions drawn regarding any association; seemingly depending upon the static measure implemented [57-60]. Static measures of navicular height are not strongly correlated with dynamic navicular motion [61] and although the FPI has been shown to correlate with dynamic measures of foot function, the strength of this correlation has varied from weak to strong [62,63]. Additionally, Barton *et al*. [64] found that dynamic measures were predictive of foot orthoses outcomes in patellofemoral pain whilst static measures of foot posture were not. Dynamic measures of foot function may well have a stronger relationship and as such may be of greater priority during clinical examination. This is explored in the accompanying dynamic review [14], which indicates plantar loading variables are risk factors for both patellofemoral pain and Achilles tendinopathy.

### Limitations and recommendations for future research

Not all studies eligible for inclusion in the current systematic review provided data suitable for meta-analysis, and obtaining this through corresponding author contact was unsuccessful in all instances. Therefore, the meta-analysis did not encompass all potentially available data, reducing confidence in its results. Complete reporting of all available data (i.e. group means, standard deviations, as well as participant numbers) in future prospective studies evaluating the potential risk of foot posture to lower limb injury is encouraged to facilitate future meta-analyses.

The average methodological quality of studies in this review was moderate, suggesting a dearth of high quality research in this area. Less than 50% of the studies included in this review [33,36,37,39,43,48] reported the reproducibility of their outcome measures; a methodological limitation that should be addressed in future research. Additionally, many studies failed to estimate their sample size based on a power calculation, cite validity and reliability data for injury determinant, or adequately adjust for covariates. Unfortunately, this further reduces the confidence in the results of our meta-analysis, but these methodological issues were taken into account during the allocation of ‘levels of evidence’ for each finding. Future studies should seek to improve upon the above limitations, as it will increase the strength of evidence than can be recommended.

Length of follow up varied greatly (eight weeks to three years), which may have an impact on injury rates and thus may affect the validity of data pooling. Future studies should seek to employ a longer duration of follow up with consideration of multiple time points to facilitate comparison between trials. An additional consideration related to data pooling is the variation in populations studied (e.g. military and running athletes), which affect loading volumes and subsequent injury risk. Nonetheless, considering the paucity of research currently available, it was felt that the pooling conducted was valuable to strengthen findings of the review. Only studies from sporting and military populations were found to be eligible and future studies investigating the impact of foot posture on injury risk in other occupational settings is warranted to determine the generalizability of these findings to other populations.

The majority of findings in this review indicating a link between foot posture and lower limb injury risk, found a more pronated foot type to be associated with increased risk. Interestingly, one of the included studies containing insufficient data for meta-analysis reported on supinated foot postures and injury risk [45], reporting that a supinated foot type based on the FPI is a risk factor for foot and ankle injury. Considering these findings, it is recommended future studies consider categorising individuals with supinated foot postures to evaluate the potential link between this foot posture and increased injury risk. This would allow similar reviews and appropriate meta-analysis to evaluate the potential link between a supinated foot posture and injury risk.

To improve the clinical applicability of results achieved, future studies should seek to describe participants in relation to both intrinsic (e.g. body mass index) and extrinsic (e.g. footwear) covariates and report risk factor statistics based on combining static foot posture data with such covariates. In comparison to nominal scaled outcome measures, continuous scaled outcome measures appear to be stronger predictors of injury development, particularly in relation to a pronated foot posture. However, because it is simpler to relate injury risk to a defined value, nominal scaled measures may be more applicable when screening for injury risk in clinical practice. Future studies that use both continuous scaled and nominal scaled data from an outcome measure (where possible) may be useful in this regard, to allow for both statistical and clinical conclusions to be reached.

## Conclusions

Strong and very limited evidence indicates that a pronated foot posture increases the risk of MTSS and patellofemoral pain, respectively. However, this relationship is of small effect, indicating that a pronated foot posture may only be a minor component of the injury risk profile for these conditions. Foot posture was not found to be associated with the risk of foot and ankle injury, bone stress reactions or a pooled group of non-specific lower limb overuse injuries, although caution with interpretation is needed here since only very limited to limited evidence exists. Of the measures used in the currently available prospective research, it appears that navicular drop and FPI can predict lower limb overuse injury, however dynamic measures of foot function may display stronger relationships with injury risk. Static measures of foot posture should be used as part of a multifactorial injury risk assessment and not considered in isolation.

## Additional files

Additional file 1
**PRISMA statement checklist.**
Additional file 2
**Search strategy.**
Additional file 3
**Epidemiological Appraisal Instrument used to rate the quality of the 21 included studies.**
Additional file 4
**Results from quality assessment using the Epidemiological Appraisal Instrument (21 included studies).**
