# Supplementary material for: Foot posture as a risk factor for lower limb overuse injury: a systematic review and meta-analysis
Source: J Foot Ankle Res. 2014 Dec 19;7:55. doi: 10.1186/s13047-014-0055-4 (PMC4282737; doi:10.1186/s13047-014-0055-4)
Supplement: Additional file 2 — Search strategy. [file 13047_2014_55_MOESM2_ESM.docx]

**Additional file 2 – Search strategy**

Ovid MEDLINE (1946 to present) and Embase Classic +Embase (1947 to present)

| 1. | exp foot/ or exp leg/ or exp foot bones/ or exp tibia/ or exp foot joint/ or exp Foot/ah, ra [Anatomy & Histology, Radiography] |
| --- | --- |
| 2. | (feet or pes or arch*).mp. [mp=ti, ab, sh, hw, tn, ot, dm, mf, dv, kw, nm, ps, rs, ui] |
| 3. | exp foot deformities/ or exp motion/ or exp joint deformities, acquired/ or exp joint instability/ |
| 4. | (dysfunction or type or posture or flat or low or high or pronat* or supinat* or arch* or cavus or planus or planovalgus or evert* or invert* or motion or structure or morphology or rearfoot).mp. [mp=ti, ab, sh, hw, tn, ot, dm, mf, dv, kw, nm, ps, rs, ui] |
| 5. | 1 or 2 |
| 6. | 3 or 4 |
| 7. | 5 and 6 |
| 8. | exp biomechanics/ |
| 9. | exp gait/ |
| 10. | locomotion/ or exp running/ or exp walking/ or motor activity/ or exp pronation/ or exp supination/ or "exp range of motion, articular"/ or motion.mp. [mp=ti, ab, sh, hw, tn, ot, dm, mf, dv, kw, nm, ps, rs, ui] |
| 11. | (kinematic* or kinetic*).mp. [mp=ti, ab, sh, hw, tn, ot, dm, mf, dv, kw, nm, ps, rs, ui] |
| 12. | 8 or 9 |
| 13. | 10 or 11 |
| 14. | 12 or 13 |
| 15. | exp Cumulative Trauma Disorders/ or "wounds and injuries"/ or exp athletic injuries/ or exp hip injuries/ or exp leg injuries/ or exp soft tissue injuries/ or exp tendon injuries/ or exp running/in [Injuries] |
| 16. | ((Oveuse or repetitive or stress or run* or sport* or impact) and (fracture* or strain or injur*)).mp. [mp=ti, ab, sh, hw, tn, ot, dm, mf, dv, kw, nm, ps, rs, ui] |
| 17. | exp Pain/ |
| 18. | 15 or 16 or 17 |
| 19. | exp Cross-Sectional Studies/ or exp Risk Factors/ or systematic review.mp. or epidemiologic studies/ or exp case-control studies/ or exp cohort studies/ or longitudinal studies/ or prospective studies.mp. [mp=ti, ab, sh, hw, tn, ot, dm, mf, dv, kw, nm, ps, rs, ui] |
| 20. | 7 or 14 |
| 21. | 18 and 19 and 20 |
| 22. | limit 21 to (english language and humans and ("prognosis (best balance of sensitivity and specificity)" or "causation-etiology (best balance of sensitivity and specificity)")) |

Studies retrieved – 8,300

SPORTDiscus (EBSCO)

| S1 | ( ((DE "FOOT") OR (DE "LEG")) OR (DE "TIBIA") ) OR TX Foot bones OR TX Foot joints |
| --- | --- |
| S2 | TX Feet OR TX Pes OR TX Arch* |
| S3 | TX Foot deformities OR TX Joint deformities OR TX Joint instability |
| S4 | TX Dysfunction OR TX Type OR TX Posture OR TX Flat OR TX Low OR TX High OR TX Pronat* OR TX Supinat* OR TX Arch* OR TX Cavus OR TX Planus OR TX Planovalgus OR TX Morpholog* |
| S5 | TX Evert* OR TX Invert* OR TX Motion OR TX Structure |
| S6 | S1 or S2 |
| S7 | S3 or S4 or S5 |
| S8 | S6 and S7 |
| S9 | DE "BIOMECHANICS" |
| S10 | DE "GAIT in humans" |
| S11 | ((((DE "LOCOMOTION") OR (DE "RUNNING")) OR (DE "PRONATION")) OR (DE "SUPINATION")) OR (DE "JOINTS -- Range of motion") |
| S12 | (DE "KINEMATICS") OR (DE "DYNAMICS") |
| S13 | S9 or S10 or S11 or S12 |
| S14 | ((((DE "OVERUSE injuries") OR (DE "SPORTS injuries")) OR (DE "SOFT tissue injuries")) OR (DE "WOUNDS & injuries")) OR (DE "RUNNING injuries") |
| S15 | TX overuse OR TX repetitive OR TX stress OR TX run OR TX sport OR TX Impact |
| S16 | TX fracture* OR TX strain OR TX injur* |
| S17 | S15 and S16 |
| S18 | DE "PAIN" |
| S19 | S14 or S17 or S18 |
| S20 | TX risk factors OR TX epidemiologic studies OR TX Case -control studies OR TX cohort studies OR TX longitudinal studies OR TX prospective studies |
| S21 | S8 or S13 |
| S22 | S19 and S20 and S21 |

Studies retrieved – 11,616

CINAHL Plus (1937 to present)

| S1 | ( ((DE "FOOT") OR (DE "LEG")) OR (DE "TIBIA") ) OR TX Foot bones OR TX Foot joints |
| --- | --- |
| S2 | TX Feet OR TX Pes OR TX Arch* |
| S3 | TX Foot deformities OR TX Joint deformities OR TX Joint instability |
| S4 | TX Dysfunction OR TX Type OR TX Posture OR TX Flat OR TX Low OR TX High OR TX Pronat* OR TX Supinat* OR TX Arch* OR TX Cavus OR TX Planus OR TX Planovalgus OR TX Morpholog* |
| S5 | TX Evert* OR TX Invert* OR TX Motion OR TX Structure |
| S6 | S1 or S2 |
| S7 | S3 or S4 or S5 |
| S8 | S6 and S7 |
| S9 | DE "BIOMECHANICS" |
| S10 | DE "GAIT in humans" |
| S11 | ((((DE "LOCOMOTION") OR (DE "RUNNING")) OR (DE "PRONATION")) OR (DE "SUPINATION")) OR (DE "JOINTS -- Range of motion") |
| S12 | (DE "KINEMATICS") OR (DE "DYNAMICS") |
| S13 | S9 or S10 or S11 or S12 |
| S14 | ((((DE "OVERUSE injuries") OR (DE "SPORTS injuries")) OR (DE "SOFT tissue injuries")) OR (DE "WOUNDS & injuries")) OR (DE "RUNNING injuries") |
| S15 | TX overuse OR TX repetitive OR TX stress OR TX run OR TX sport OR TX Impact |
| S16 | TX fracture* OR TX strain OR TX injur* |
| S17 | S15 and S16 |
| S18 | DE "PAIN" |
| S19 | S14 or S17 or S18 |
| S20 | TX risk factors OR TX epidemiologic studies OR TX Case -control studies OR TX cohort studies OR TX longitudinal studies OR TX prospective studies |
| S21 | S8 or S13 |
| S22 | S19 and S20 and S21 |

Studies retrieved – 13,602
