# Supplementary material for: Foot posture as a risk factor for lower limb overuse injury: a systematic review and meta-analysis
Source: J Foot Ankle Res. 2014 Dec 19;7:55. doi: 10.1186/s13047-014-0055-4 (PMC4282737; doi:10.1186/s13047-014-0055-4)
Supplement: Additional file 3 — Epidemiological Appraisal Instrument used to rate the quality of the 21 included studies. [file 13047_2014_55_MOESM3_ESM.pdf]

Additional file 3 - Epidemiological appraisal instrument

	Methodological quality assessment	Yes/High 2	Partial/Moderate 1	No/Low 0	Not applicable (N/A)	Unable to determine (UTD) 0	Tips	Comments
				**Section (i) reporting**				
Hypothesis/aim/objective	**1. Is the hypothesis or aim or objective of the study clearly described?**	The objective is clearly stated in one or two statements in the introduction (Might include but not limited to- study design - prospective cohort/study/design, adequate discription of- how foot posture is investigated and injury/s of interest so that the study can be replicated)	There is sufficient information to be able to infer the objective in the introduction	The study objective is not described in the introduction and there is insufficient information provided to even 'infer'				
Exposure	**2. Are all the risk factor variable(s) related to foot posture clearly described?**	The definitions of all risk factor variables related to foot posture are referenced to a clear description (e.g. Navicular Drop + description) if described with reference Yes (pending)	The definitions of all risk factor variable related to foot posture are not clearly described, but sufficient information is provided for the reader to understand the intent (e.g. Navicular drop with poor description)<br>OR<br>Some, but not all, of the foot posture relatd risk factor variables are described (i.e. multiple foot posture risk factors are investigated, but only individual risk factors are described)	No mention of definition of risk factor variables related to foot posture (e.g. Navicular drop without description)			"All risk factor variables" may only include a single foot risk variable i.e. individual measure of foot posture<br>Non-foot posture related risk factor variables do not need to be analysed in this question	
Outcome	**3. Is injury clearly described?**	The definition of all injuries are referenced to a clear description (e.g. Injury + adequate discription of how injury will be determined and measured)	The definition of all injuries are not clearly described, but sufficient information is provided for the reader to understand the intent (i.e. Injury with poor description of how injury will be determined and measured)	No mention of definition of injury (e.g. Injury without description of how injury will be determined or measured)				
Study Design	**4. Is the study design clearly described?**	The study design is clearly described using the following terminology (or similar):<br>**prospective cohort**	Study design has to be inferred (i.e. prospective study, no use of the word cohort or group was followed over time etc.)	No mention of study design (i.e. no description)				
Study Population	**5. Is the source of subject population (including sampling frame) clearly described?**	The following details are clearly described:<br>1. Geographic location and/or setting- Australia, Uk or setting<br>2. Type of list of potential subjects- where were subjects recruited from e.g. military personal, running club<br>3. Time frame of initial participant data collection- eg participants were collected over x weeks (needs to be a specific measure of time)	One or more of the following details reported:<br>1. Geographic location and/or setting- Australia, Uk or setting<br>2. Type of list of potential subjects- where were subjects recruited from e.g. military personal, running club<br>3. Time frame of initial participant data collection- eg participants were collected over x weeks (needs to be a specific measure of time)	No mention of source population				
	**6. Are the eligibility criteria for subject selection clearly described?**	Inclusion and/or exclusion criteria of the study population are clearly described in a few sentences	Inclusion and/or exclusion criteria are mentioned but not clearly described<br>OR<br>Inclusion of the entire eligible population must be inferred	Inclusion and/or exclusion criteria are not mentioned or described				
	**7. Are the participation rate(s) reported? Are ascertainments of record availability described?**	Participation rates are reported for the overall population<br>OR<br>Subject numbers are clearly given so that participation rates may be calculated<br>e.g. Screened this many and used this many	Participation rates have to be inferred<br>OR<br>Are reported for some but not all participants	Participation rates not reported<br>OR<br>Any other situation not described in YES or PARTIAL	N/A for national surveys (i.e. census data)		Participant Rate = (number of participants eligible - participant not willing to participate)/ number of participants<br><br>Different from subjects lost, subjects lost referred to the subjects that dropped out after initial data was collected	
	**8. Are the characteristics of study participants described?**	Subject characteristics are adequately described for the overall population (at least 2 of the following: age - mean or range, gender, ethnicity)	Subject characteristics are not adequately described (only one of the following: age - mean or range, gender, ethnicity)<br>OR<br>Subject characteristics have to be calculated from data tables	No mention of subject characteristics				
	**9. Have the characteristics of subjects lost after entry into the study OR subjects not participating been described? Have the details of unavailable records been described.**	Characteristics of subjects lost or details are described in an equally detailed way to question 8 - "Yes"<br>OR<br>There are no losses or losses are so small that findings would be unaffected (less than 10% for each group or 10% of overall population)	The characteristics are poorly described in an equally detailed way to question 8 - "Partial"<br>OR<br>Losses in all groups are less than 20%	Characteristics of subjects lost/unavailable records are not reported<br>OR<br>Losses in all groups are greater than 20%			Subjects lost = participants dropped out after initial data was collected	
	**10. Have all important adverse effects been reported that may be consequences of the interventions?**				Cohort study (no intervention)			
	**11. Are the important intrinsic risk factors (confounders and covariates) for injury been described in terms of individual variables?**	All intrinsic risk factors (all from the green list below) have been listed and described	Some (but not all) intrinsic risk factors (one or more from the green list below) have been listed and described	No mention of any co-variates or confounders				
	**12. Are the important extrinsic risk factors (confounders and covariates) for injury been described in terms of individual variables?**	All extrinsic risk factors (all from the red list below) have been listed and described	Some (but not all) extrinsic risk factors (one or more from the red list below) have been listed and described	No mention of any co-variates or confounders				

Statistical Tests	**13. Are the statistical methods clearly described?**	All relevant statistical tests are listed and clearly described (i.e. Chi squared, multiple logistic analysis etc.)<br>AND<br>All confounders and co-variates **(all from red and green the list below)** did not statistically affect the injury rate	Some but not all relevant statistical tests are listed and clearly described (eg Chi squared, multiple logistic analysis etc.)<br>AND<br>Some but not all confounders and co-variates that were investigated in the study **(1 or more from the red and green list below)** did not statistically affect the injury rate	No mention of any statistical test<br>OR<br>No confonfounders investigated were reported<br>OR<br>Any other situation not listed under "Yes" or "Partial"				
Results	**14. Are the main findings of the study clearly described?**	Basic data for all foot posture related risk factor variables and all injury outcome are reported so that the reader can check the major analyses and conclusions	Basic data for foot posture related risk factor variables and injury outcome are reported for some (but not all) groups	No mention of any outcome or risk factor data<br>OR<br>Any other situation not listed under 'Yes' or 'Partial'			"All risk factor variables" may only include a single foot risk variable ie. individual measure of foot posture Non-foot posture related risk factor variables do not need to be analysed in this question.	
	**15. Does the study provide estimates of the random variability in the data for the outcome of interest (i.e. confidence intervals, standard deviations)?**	For normal data, confidence intervals or standard errors for all outcomes (injury) or foot posture related risk factors<br>OR<br>The inner quartile range for non-normally distributed data for all outcomes (injury) or foot posture related risk factors	Estimates of random variability (ie. Confidence intervals etc) are reported for some (but not all) outcomes (injury) and foot posture related risk factor variables<br>Only provides results for overall population	No mention of any estimates of random variability OR any other situation not described under 'Yes' or 'Partial'				
	**16. Does the study provide estimates of statistical parameters (eg. Regression coefficients or parameter estimates such as odds ratio)? Ie magnitude of significance**	Estimates are reported for all different parameters (injury or foot posture risk factor variables)<br>i.e. Odds/risk ratio or effect size for the development of injury for all foot posture risk factors investigated	Estimates are reported for some (but not all) groups (designs specifying groups)<br>OR<br>Estimates are only provided for some parameters<br>OR<br>Only provides results for overall population	No mention of any estimates<br>OR<br>Any other situation not listed under 'Yes' or 'Partial'			"All risk factor variables" may only include a single foot risk variable ie. individual measure of foot posture Non-foot posture related risk factor variables do not need to be analysed in this question	
	**17. Are sample size calculations performed and reported?**	The study has performed an analysis where calculations were used and reported in the paper to determine how many participants were required for the calculation of all of the following:<br>- effect size<br>- type I or II errors<br>- number of confounders	Calculations are performed with details reported for one or more of the following:<br>- effect size<br>- type I or II errors<br>- number of confounders.	Calculations are commented to have been performed but no details reported<br>OR<br>No mention of any attempt to perform calculations (sample of convenience)				
				**Section (ii) subject/record selection**				
Group Comparability	**18. Is the comparison/reference group comparable to the exposed/intervention/case group?**	All groups are drawn from the same eligible population	Control/comparison groups are not drawn from the same eligible population, but recruited from similar populations elsewhere	Controls are not used OR national controls or external groups are used	Studies performing correlations within one group	Insufficient details	Prospective cohort studies would be considered **N/A** as there has been no initial separation of the sample group<br>A score of **N/A** would be considered for studies that have grouped participants after injury is recorded<br>If there is **initial separation** based on foot posture, these groups should be appraised in a similar way to a case-control study	
Participation Rate	**19. Is the particpation rate adequate? Is the ascertainment of record availability adequate?**	Participation rate or record availability >80% (INCLUDING convenience samples where 100% participation rate is reported)<br>e.g. A study would be considered yes if the study had a population of 1000 and only recruited 900 (90%)	Participation rate ≥50%	Participation rate <50%	N/A for national surveys	Insufficient details	Participant Rate = (number of participants eligible - participant not willing to participate)/ number of participants	
Time period	**20. Are the study subjects from different groups OR the cohort recruited over the same period of time?**	Within 6 months<br>Study needs to mention that participants were recruited within 6 months of each other prior to prospective evaluation	Less than a year<br>Study needs to mention that participants were recruited within 1 year of each other prior to prospective evaluation	More than a year<br>Study needs to mention that participants were recruited greater than 1 year of each other prior to prospective evaluation	Cross-sectional designs utilizing overall population	Insufficient details	The term "different groups" for prospective cohort subjects only applies if foot posture has been used to seperate the cohort initially and these groups are recruited over different time periods	
Subject losses	**21. Are subject losses or unavailable records after entry into the study taken into account?**	Characteristics of non-responders or unavailable records are described in identical way (Question 9 - "Yes") and are not significantly different from those of the study participants or available records<br>OR<br>Losses <10%	Characteritics of non-responders or unavailable records are described in identical way to those of the participating subjects or available records (Question 9 - "Yes") BUT no mention of statistical differences among groups<br>OR<br>Losses <20%	No mention or poor discription of non responder characteristics and/or unavailable records (Question 9 - "Partial" or "No")<br>OR<br>Any other situation not listed under 'Yes' or 'Partial'	N/A for national survey OR convenience samples where 100% participation rate is reported or inferred		Losses refer to subjects that were lost after initial data was collected	
Type of Cases	**22. Are newly incident cases taken into account?**				Cohort observational study (no intervention)			
Randomisation	**23. Are the study subjects randomised to groups?**				Cohort observational study (no intervention)			
	**24. Is the randomised assignment to groups concealed from both subjects and observers until recruitment is complete irrevocable?**				Cohort observational study (no intervention)			

Section (iii) measurement quality								
Measurement quality	**25. Are measurement methods for risk factor variables reliable?**	Reliability > 0.70 for <u>all</u> foot posture risk factor variables<br>AND<br>Study needs to use the word "reliability" (or similar) followed by the reference or determined value<br>- Determined there own reliability<br>- Reference of reliability (Yes - pending)	Reliability ≥0.40 for <u>all foot posture</u> risk factor variables<br>AND<br>Study needs to use the word "reliability" (or similar) followed by the reference or determined value<br>- Determined there own reliability<br>- Reference of reliability (Partial - pending)	Reliability <0.40 for at least one foot posture risk factor variable<br>OR<br>Poor documentation of reliability from prior work from the published literature - study uses the word "reliability" (or similar) but does not present determined value or reference		No mention of reliability of risk factor variables	"All risk factor variables" may only include a single foot risk variable ie. individual measure of foot posture<br>Non-foot posture related risk factor variables do not need to be analysed in this question	
	**26. Are measurement methods for risk factor variables valid?**	Foot posture risk factor variable is the <u>gold standard</u> (see list below)<br>OR<br>Validity >0.70 for all foot posture risk factors that are not the gold standard<br>OR<br>Detailed documentation of validity from prior work from the published literature - study needs to use the word "validity" (or similar) followed by a reference or determined value	Validity ≥0.40 for all foot posture risk factor variables that are not the gold standard<br>OR<br>Somewhat detailed documentation of validity from prior work from the published literature - study needs to use the word "validity" (or similar) followed by a reference or determined value	Validity <0.40 for at least one foot posture risk factor variable that is not the gold standard<br>OR<br>Poor documentation of validity from prior work from the published literature - study uses the word "validity" (or similar) but does not present determined value or reference		No mention of validity of risk factor variables		
	**27. Are the methods of assessing the risk factor variables standard for all participants?**	Measurement methods of foot posture related risk factors are comparable for all participants<br>Study must state that all subjects were measured in the same way, by the same investigator	Some differences in measurement methods of foot posture related risk factors:<br>- Same measurement technique used on all participants BUT - different raters were used to measure variable<br>OR<br>-Not stated that all measurements were performed by the same investigator	Different measurement methods<br>-Different methods for measuring foot posture were used on different participants		Insufficient details		
	**28. Is the measurement conducted at a time prior to injury?**	Measurement occured at a time prior to injury (Study does not need to specificy time)		Measurement did no occur at a time prior to injury<br>OR<br>No comment that participants were measured prior to observation			Study does not have to specify time	
Blind measurement	**29. Are the observers blinded to subject groupings/disease status when the risk factor assessment was made?**	Observers are <u>truly</u> blinded to group status while conducting assessment (i.e. by design observers are blinded to subject grouping AND there is no way the observers can figure out subject groupings)	Observers are <u>not truly</u> blinded (i.e. by design the observers are blinded to subject grouping; however, you may infer that it is possible for the observers to figure out subject groupings)	Observers are <u>not</u> blinded	Cross-sectional designs utilizing overall population<br>A score of N/A would be considered for studies that have grouped participants after injury is recorded not at baseline	Insufficient details	For single group cohort studies, choose N/A.<br>**A score of N/A would be considered for studies that have grouped participants after injury is recorded not at baseline**<br>For cohort studies that split groups at <u>baseline</u> based on risk factors (e.g. Supinated vs. Pronated), choose criteria based on whether the observers knew which group subjects were allocated to at baseline (i.e. prior to reporting injury)	
	**30. Are the subjects blinded to their grouping when the exposure was made?**	Subjects are <u>truly</u> blinded to exposure/intervention and comparison groups (i.e. by design, the subjects are blinded to their group AND there is no way that the subjects are aware of their grouping)	Subjects are <u>not truly</u> blinded (i.e. by design, the subjects are blinded to their group; however , you may infer that it is possible for the subjects to figure out which group they are in)	Subjects are <u>not</u> blinded	Cross-sectional design utilising only overall population without specific groups<br>A score of N/A would be considered for studies that have grouped participants after injury is recorded not at baseline	Insuffiecient details	For single group cohort studies, choose N/A.<br>**A score of N/A would be considered for studies that have grouped participants after injury is recorded not at baseline**<br>For cohort studies that split groups at <u>baseline</u> based on risk factors (e.g. Supinated vs. Pronated), choose criteria based on whether the observers knew which group subjects were allocated to at baseline (i.e. prior to reporting injury)	
Outcome	**31. Is reliability described for the measurement of the injury of interest?**	Reliability > 0.70 for outcome (injury) variables<br>And<br>Study needs to use the word "reliability" (or similar) followed by the reference or determined value<br>- Determined there own reliability<br>- Reference of reliability (Yes - pending)	Reliability ≥0.40 for outcome (injury) variables<br>And<br>Study needs to use the word "reliability" (or similar)followed by the reference or determined value<br>- Determined there own reliability<br>- Reference of reliability (Partial - pending)	Reliability <0.40 for outcome (injury) variables<br>OR<br>Poor documentation of reliability from prior work from the published literature - study uses the word "reliability" (or similar) but does not present determined value or refernce		No mention of reliability of outcome variables		
	**32. Is validity described for the measurement of the injury of interest?**	VALID: Outcome measure is the gold standard<br>OR<br>Validity > 0.70 if outcome measure is not the gold standard<br>OR<br>Detailed documentation of validity from prior work from the published literature - study needs to use the word "validity" (or similar) followed by a reference or determined value	SOMEWHAT VALID: Validity ≥0.40 (if outcome measure is not the gold standard)<br>OR<br>Somewhat detailed documentation of validity from prior work from the published literature - study needs to use the word "validity" (or similar) followed by a reference or determined value	POOR: Validity <0.40 (if outcome measure is not the gold standard)<br>OR<br>Poor documentation of validity from prior work from the published literature - study uses the word "validity" (or similar) but does not present determined value or reference		No mention of validity of outcome variables		
	**33. Are the methods of assessing the outcome variables standard for all participants?**	Outcome assessment methods are comparable for <u>all</u> participants (example: Injury investigation is performed by the same observer and in the same way for all participants)	Some differences (example: Injury investigations are performed by different people in the same way for all participants not repeated on the same participant)	Different assessment methods for determining injury for different participants		Insufficient details		
	**34. Are the observations taken over the same time for all groups?**	Within 6 months<br>(e.g. participant 1 followed over - 2 years, participant 2 followed over - 2.4 years	Less than a year	More than a year	Cross-sectional designs utilizing overall population *OR studies utilizing cases only with no control group*	Insufficient details	Needs to be stated that all subjects were followed for x months not using phrases such as "followed for one season"	

				**Section (iv) data analysis**				
Covariates and confounders	**35. Is prior history of disease and/or symptoms collected and included in the analysis?**	Data on disease/symptom history collected and accounted for in the statistical analyses	Data on disease/symptom history collected and not accounted for in the analysis	Not collected or accounted for in the statistical analyses	The study excluded participants based on prior history<br>OR<br>Proportionate design<br>OR<br>Outcomes for which history of disease is irrelevant such as cancer<br>OR<br>Matched at the design stage and excluded prior to start in the study<br>OR<br>Study reported that there was no injury in the group			
	**36. Is there adequate adjustment for intrinsic variables?**	Adjustments are made for all intrinsic covariates and confounders (all from the green list below) in the final analysis with the use of statistical techniques such as multivariate analysis and/or statistical design such as matching	Only some of the intrinsic variables are considered in the analysis (only one or more from the green list below)	No mention of intrinsic covariates or confounders OR not adjusted for in any statistical tests				
	**37. Is there adequate adjustment for extrinsic variables?**	Adjustments are made for all extrinsic covariates and confounders (all from the red list below) in the final analysis with the use of statistical techniques such as multivariate analysis and/or statistical design such as matching	Only some of the extrinsic variables are considered in the analysis (only one or more from the red list below)	No mention of extrinsic covariates or confounders OR not adjusted for in any statistical tests				
	**38. Is the minimum follow-up time since initial assessment sufficient enough to detect a relationship between the risk factor and injury?**	Follow-up time is ≥ 12 weeks	Follow-up time is between 6-11 weeks	Follow-up time is ≤ 5 weeks<br>OR<br>Not assessed		Insuffiecient details	Will depend on intensity of program, injury of interest and other cavariates	
	**39. Does the analysis adjust for different length of follow up, of subjects in cohort studies; is the time period between assessment and injury the same for cases and controls?**	Follow-up time is the same for all subjects<br>OR<br>Adjustment is made for all subjects in the analysis	Adjustment is not made for all subjects in the analysis	No adjustment is made for any of the subjects, that is, studies where differences are ingnored should be answered 'no'		Insuffiecient details	Important to note question 20 and whether all participants were the same	
	**40. Is injury data reported by different levels of assessed risk factors? (based on discontinuous or continuous measure of foot posture)**	For discontinuous variables outcome data are reported for all outcome variables for at least three levels of foot posture related risk factors (supination, pronation and neutral [or similar])<br>OR<br>For continuous measures of foot posture there is correlation between the injured and non injured population for all foot posture related risk factor variables	For discontinuous variables outcome data are reported for some but not all outcome variables for at least two levels of foot posture related risk factors (A minimum of two of the following; supination, pronation and neutral [or similar])<br>OR<br>For continuous measures there is correlation reported for some but not all foot posture related risk factors	Data are presented as present/absent for risk factor variables OR any other situation not described for "Yes" and "Partial"		Insufficient details	E.g. Injury rates reported for different levels of foot posture (pronated, neutral, supinated [or similar cavus etc])	
	**41. Are the data on injuries reported by subgroups of subjects other than foot posture?**	Analyses are reported for two or more subgroups of subjects other than foot posture (eg discontinuous measures- gender etc OR for continuous measures - age, height, weight etc.)<br>AND<br>Reported for all injuries	Analyses are reported for one subgroups of subjects other than foot posture (eg discontinuous measures-gender etc OR for continuous measures - age, height, weight etc.)<br>AND<br>Reported for some injuries	Not reported for any subgroups<br>Not reported for any injury				
				**Section (v) generalisation of results**				
Generalization of results	**42. Can the study results be applied to the eligible population?**	Participation rate >80% AND characteristics of non-responders are not significantly different	Participation rate ≥50% AND characteristics of non-responders are not significantly different	Participation rate or available records <50% OR characteristics of non-responders are significantly different	N/A for national sample OR convenience samples where 100% participation rate is reported or inferred	Participation rate not reported OR non-responders not described	Review questions 7-9	
	**43. Can the study results be applied to other relevant populations?**	The results are expected to apply to other relevant groups; study sample is taken by random sampling from the general population (eg electoral role)	The results are somewhat applicable to other relevant groups; study sample taken by convenience sampling	Results are not applicable to other relevant groups; biased sample of individuals seeking treatment for foot problems OR cases are studied with no control group		Sampling method not reported		

Disagreements
**Risk factor variable**

**Risk factor variable**	**Gold standard**	**Test for validity**
Pronation	no gold standard	Attempt to validate using another reliable tool (e.g. foot posture index)?
Footwear factors	no gold standard	
Plantar pressures	no gold standard	

LIST OF COVARIATES

Extrinsic Covariates	Footwear	
	Ankle bracing and orthotic use	
	Playing/running surface	
	Sport type	
	Skill level	

Intrinsic Covariates	Age	
	Sex	
	Previous Injury	
	Body Size	
	Joint laxity	If the study is interested in a specific joint injury – need to comment on the specific joints laxity<br><br>E.g. study interested in foot postures influence of knee pain – study has to investigate knee joint laxity
	Joint ROM	If the study is interested in a specific joint injury – need to comment on specific joint ROM<br>E.g. study interested in foot postures influence of knee pain – study has to investigate knee joint ROM.
