# Supplementary material for: Foot posture as a risk factor for lower limb overuse injury: a systematic review and meta-analysis
Source: J Foot Ankle Res. 2014 Dec 19;7:55. doi: 10.1186/s13047-014-0055-4 (PMC4282737; doi:10.1186/s13047-014-0055-4)
Supplement: Additional file 4 — Results from quality assessment using the Epidemiological Appraisal Instrument (21 included studies). [file 13047_2014_55_MOESM4_ESM.pdf]

Additional file 4. Results from quality assessment using the Epidemiological Appraisal Instrument (21 included studies)

Black shading = "yes", Grey shading = "partial", White (no shading) = "no" or "unable to determine", . = "not applicable"

Study ID	Bennett et al. 2001	Yates et al. 2004	Burne et al. 2004	Willems et al. 2006	Reinking et al. 2006	Reinking et al. 2007	Plisky et al. 2007	Hubbard et al. 2009	Bennett et al. 2012	Yagi et al. 2014		Studies scoring yes (x/21)
Q1. Reported study aim/objective clearly												20
Q2. Risk factors related to foot posture are clearly defined												13
Q3. Overuse injury clearly defined												12
Q4. Reported study design												16
Q5. Reported subject population												9
Q6. Reported includion criteria												8
Q7. Reported participation rate												13
Q8. Reported participant characteristics												16
Q9. Reported participant lost characteristics												16
Q10. Reported adverse reactions	.	.	.	.	.	.	.	.	.	.		N/A
Q11. Reported intrinsic risk factors												3
Q12. Reported extrinsic risk factors												3
Q13. Reported statistical methods												17
Q14. Reported all basic data												20
Q15. Reported variability in data												17
Q16. Reported statistical parameters												15
Q17. Sample size calculations												5
Q18. Comparatibility of case/control groups	.	.	.	.	.	.	.	.	.	.		N/A
Q19. Adequate participation rate												18
Q20. Recruitment period	.	.	.	.	.	.	.	.	.	.		N/A
Q21. Non-responder characteristics described												15
Q22. New incident case analysis	.	.	.	.	.	.	.	.	.	.		N/A
Q23. Subject randomisation	.	.	.	.	.	.	.	.	.	.		N/A
Q24. Randomisation concealment	.	.	.	.	.	.	.	.	.	.		N/A
Q25. Reported reliability of foot posture risk factor measurement												8
Q26. Reported validity of foot posture risk factor measurement												3
Q27. Reported standardisation of foot posture risk factor measurement												4
Q28. Was foot posture measurement conducted prior to injury												21
Q29. Observer blinding	.	.	.	.	.	.	.	.	.	.		N/A
Q30. Subject blinding	.	.	.	.	.	.	.	.	.	.		N/A
Q.31 Reported reliability of overuse injury measurement												0
Q.32 Reported validity of overuse injury measurement												0
Q33. Reported standardisation of overuse injury measurement												8
Q34. Observation time comparability												16
Q35. Reported prior disease/history												4
Q36. Adjustment for intrinsic variables												3

Q37. Adjustment for extrinsic variables												1
Q38. Is follow up time adequate												16
Q39. Follow up time differences												19
Q40. Reported data ≥3 levels of associated factors												18
Q41. Reported data for subgroups of participants (e.g. by gender or age)												13
Q42. Generalisability of results to study population (participation rate)												20
Q43. Generalisability of results to other populations (random sampling)												1
Overall quality score (range 0 to 2)	1.2	1.3	1.4	1.3	1.5	1.2	1.7	1.1	1.4	1.3		

Study ID	Hetresoni et al. 2006	Thjis et al. 2008	Boling et al. 2009	Beynonn et al. 2001	Cain et al. 2007	Winfield et al. 1997	Kaufman et al. 1999	Burns et al. 2005	Rauh et al. 2010	Buist et al. 2010	Nielsen et al. 2013	Studies scoring yes (x/21)
Q1. Reported study aim/objective clearly												20
Q2. Risk factors related to foot posture are clearly defined												13
Q3. Overuse injury clearly defined												12
Q4. Reported study design												16
Q5. Reported subject population												9
Q6. Reported includion criteria												8
Q7. Reported participation rate												13
Q8. Reported participant characteristics												16
Q9. Reported participant lost characteristics												16
Q10. Reported adverse reactions	•	•	•	•	•	•	•	•	•	•	•	N/A
Q11. Reported intrinsic risk factors												3
Q12. Reported extrinsic risk factors												3
Q13. Reported statistical methods												17
Q14. Reported all basic data												20
Q15. Reported variability in data												17
Q16. Reported statistical parameters												15
Q17. Sample size calculations												5
Q18. Comparatibility of case/control groups	•	•	•	•	•	•	•	•	•	•	•	N/A
Q19. Adequate participation rate												18
Q20. Recruitment period	•	•	•	•	•	•	•	•	•	•	•	N/A
Q21. Non-responder characteristics described												15
Q22. New incident case analysis	•	•	•	•	•	•	•	•	•	•	•	N/A
Q23. Subject randomisation	•	•	•	•	•	•	•	•	•	•	•	N/A
Q24. Randomisation concealment	•	•	•	•	•	•	•	•	•	•	•	N/A
Q25. Reported reliability of foot posture risk factor measurement												8
Q26. Reported validity of foot posture risk factor measurement												3
Q27. Reported standardisation of foot posture risk factor measurement												4
Q28. Was foot posture measurement conducted prior to injury												21
Q29. Observer blinding	•	•	•	•	•	•	•	•	•	•	•	N/A
Q30. Subject blinding	•	•	•	•	•	•	•	•	•	•	•	N/A
Q.31 Reported reliability of overuse injury measurement												0

Q.32 Reported validity of overuse injury measurement												0
Q33. Reported standardisation of overuse injury measurement												8
Q34. Observation time comparability												16
Q35. Reported prior disease/history												4
Q36. Adjustment for intrinsic variables												3
Q37. Adjustment for extrinsic variables												1
Q38. Is follow up time adequate												16
Q39. Follow up time differences												19
Q40. Reported data ≥3 levels of associated factors												18
Q41. Reported data for subgroups of participants (e.g. by gender or age)												13
Q42. Generalisability of results to study population (participation rate)												20
Q43. Generalisability of results to other populations (random sampling)												1
Overall quality score (range 0 to 2)	1	1.2	1.1	1.3	1.3	0.8	1.4	1.5	1.1	1.3	1.5	
